# Gene Sequence Based Clustering Assists in Dereplication of *Pseudoalteromonas luteoviolacea* Strains with Identical Inhibitory Activity and Antibiotic Production 

**DOI:** 10.3390/md10081729

**Published:** 2012-08-15

**Authors:** Nikolaj G. Vynne, Maria Mansson, Lone Gram

**Affiliations:** 1 National Food Institute, Technical University of Denmark, Søltofts Plads bldg 221, DK-2800 Kgs. Lyngby, Denmark; Email: gram@food.dtu.dk; 2 Center for Microbial Biotechnology, Department of Systems Biology, Technical University of Denmark, Søltofts Plads bldg 221, DK-2800 Kgs. Lyngby, Denmark; Email: maj@bio.dtu.dk

**Keywords:** *Pseudoalteromonas luteoviolacea*, violacein, indolmycin, pentabromopseudilin, biodiscovery

## Abstract

Some microbial species are chemically homogenous, and the same secondary metabolites are found in all strains. In contrast, we previously found that five strains of *P. luteoviolacea* were closely related by 16S rRNA gene sequence but produced two different antibiotic profiles. The purpose of the present study was to determine whether such bioactivity differences could be linked to genotypes allowing methods from phylogenetic analysis to aid in selection of strains for biodiscovery. Thirteen *P. luteoviolacea* strains divided into three chemotypes based on production of known antibiotics and four antibacterial profiles based on inhibition assays against *Vibrio anguillarum* and *Staphylococcus aureus*. To determine whether chemotype and inhibition profile are reflected by phylogenetic clustering we sequenced 16S rRNA, *gyrB* and *recA* genes. Clustering based on 16S rRNA gene sequences alone showed little correlation to chemotypes and inhibition profiles, while clustering based on concatenated 16S rRNA, *gyrB*, and *recA* gene sequences resulted in three clusters, two of which uniformly consisted of strains of identical chemotype and inhibition profile. A major time sink in natural products discovery is the effort spent rediscovering known compounds, and this study indicates that phylogeny clustering of bioactive species has the potential to be a useful dereplication tool in biodiscovery efforts.

## 1. Introduction

Many antibiotics used in treatment of infectious disease are of natural product origin, and despite high hopes for new drug discovery strategies, alternative approaches to drug discovery such as combinatorial chemistry has failed to adequately supply the drug pipeline [1]. Therefore, we must revert to discovery of novel natural products capable of inhibiting or killing pathogenic bacteria [2], and screening of microorganisms [3] from extreme or underexplored environments [4] could be a promising approach. The marine environment is still considered an underexplored source of novel antimicrobial compounds and marine microorganisms are viewed as a potential source of novel antibiotic compounds [5,6,7]. Indeed, marine bacteria belonging to the Cyanobacteria [8], Actinobacteria [9], the *Roseobacter* clade [10,11], and the *Pseudoalteromonas* genus [12,13] produce compounds with interesting pharmacological properties. 

Members of the genus *Pseudoalteromonas* are ubiquitous in the marine environment [14] and form two groups supported by analysis of 16S rRNA gene sequences [15]. One group predominantly consists of non-pigmented pelagic bacteria with low or no antibacterial activity while most species in the other group are pigmented, antagonistic, and found as colonizers marine biotic surfaces, thus pigmented Pseudoalteromonads represent a promising target for biodiscovery efforts.

One of the main challenges in natural products discovery is the effort squandered rediscovering known compounds [16], hence so called “dereplication” strategies to reduce the degree of rediscovery prior to purification of compounds and structure elucidation steps are of outmost importance [17,18,19]. One such strategy is early stage dereplication informed by microbial systematics [20,21,22]. Some bacterial species are chemically very homogeneous and all strains produce the same biologically active secondary metabolites as is seen for production of tropodithietic acid in *Phaeobacter gallaeciencis* or *Ruegeria mobilis* [23] or salinisporamide A in *Salinispora tropica* [24]. This makes early dereplication of such strains of high value in biodiscovery to reduce discoveries of one compound from several strains. Add to this that novel bacterial diversity likely also represents a reservoir for novel chemistry and it is clear why bacterial systematics may play a role in natural product discovery. Indeed, Goodfellow and Fiedler [25] recently pointed out that screening of a taxonomically dereplicated collection of Actinobacteria led to discovery of a high number of novel compounds relative to the strain throughput. Hence, it may be possible to apply knowledge of bacterial systematics and taxonomy as a guide for efficient biodiscovery within bacteria. We previously reported [26] that five *Pseudoalteromonas luteoviolacea* strains with nearly identical 16S rRNA gene sequences produced two combinations of the three antibacterial compounds violacein [27], indolmycin [28], and pentabromopseudilin [29]. Hence, *P. luteoviolacea* was suitable for investigating relations between bacterial systematics and production of bioactive secondary metabolites at the infra-species level.

The aim of the present study was to determine if strain differences in production of antibacterial compounds by *Pseudoalteromonas luteoviolacea* were linked to systematic groups which may point to phylogenetic analyses as a tool in biodiscovery. 16S rRNA, *gyrB*, and *recA* genes of 13 *P. luteoviolacea* strains were sequenced and used in sub-typing. Coupled with information on antibiosis obtained from agar based inhibition assays, we found that the combination of screening for inhibition and sub-typing by phylogenetic analysis would allow selection of representative bioactive strains within this collection of *P. luteoviolacea* strains and hence could represent an approach for dereplication of *Pseudoalteromonas* strains in small-molecule biodiscovery programs.

## 2. Results and Discussion

### 2.1. *Pseudoalteromonas luteoviolacea* Production of Antibacterial Compounds and Antibacterial Activity

All 13 strains produced the purple antibacterial pigment, violacein (Table 1). Five strains produced pentabromopseudilin (chemotype 1), three strains produced indolmycin (chemotype 2) and four strains did not produce other known antibiotics (chemotype 3). No strain produced both indolmycin and pentabromopseudilin. Hence, the sub-division previously seen with respect to pentabromopseudilin and indolmycin was confirmed in a larger selection of strains. We have attempted to further broaden our collection; however, we were unable to do so as *P. luteoviolacea* under certain conditions is autoinhibitory [30] and several laboratories no longer had stock cultures. 

Despite the violacein production by all strains, just 11 of the 13 strains inhibited *V. anguillarum* and *S. aureus* in the live cell assay (Table 1). This indicates that violacein is not a major antibacterial compound under these conditions. Instead, violacein has been suggested to act as a cell-associated anti-predation compound [31] which is more in line with its low solubility in water and distinct cell-association and offers an ecological role for this compound which may explain the ubiquitous production within strains of *P. luteoviolacea*. Sterile filtered culture supernatants were tested in well diffusion agar assays. Sterile filtered supernatants were antibacterial only if harvested from strains producing either pentabromopseudilin (6 strains, inhibition profile A) or indolmycin (3 strains, inhibition profile B). Both indolmycin and pentabromopseudilin containing supernatants inhibited *S. aureus*, whereas only indolmycin containing supernatants inhibited the Gram-negative *V. anguillarum* (Table 1). This is in agreement with previous studies describing pentabromopseudilin as a compound targeting Gram-positive bacteria, whereas indolmycin, previously only isolated from *Streptomyces* species [32,33], inhibits both Gram-positive and Gram-negative bacteria and is very potent against staphylococci [34]. The supernatants from the remaining four strains were not inhibitory against either of the target organisms and these four strains produced neither indolmycin nor pentabromopseudilin. Discrepancies among live cell and sterile supernatant inhibition profiles (inhibition profile C) suggest that additional antibiotic compounds may be produced, for instance macromolecular antibiotics such as L-amino acid oxidases which are produced by some *P. luteoviolacea* strains [35]. 

**Table 1:** Inhibitory activity of *P. luteoviolacea* strains in the live cell and sterile filtered supernatant well diffusion agar assays and production of three known antibacterial compounds (PBP = pentabromopseudilin) in marine minimal medium cultures. The production of antibacterial compounds was determined by qualitative LC-MS analysis. *V. ang.* = *Vibrio anguillarum*; x: Inhibition or compound production, respectively; -: No inhibition or compound production.

	Live cell inhibition			Supernatant inhibition			Antibacterial compounds produced					Inhibition profile	Genotype *
Strain	*V. ang.*	*S. aureus*		*V. ang.*	*S. aureus*		Violacein	PBP	Indolmycin		Chemotype		
2ta16	x	x		-	x		x	x	-		1	A	I
NCIMB 1944	x	x		-	x		x	x	-		1	A	I
DSM6061 ^T^	x	x		-	x		x	x	-		1	A	I
CPMOR-2	x	x		-	x		x	x	-		1	A	I
S2607	x	x		-	x		x	x	-		1	A	III
S4060-1	x	x		-	x		x	x	-		1	A	III
S4047-1	x	x		x	x		x	-	x		2	B	II
S4054 WT	x	x		x	x		x	-	x		2	B	II
CPMOR-1	x	x		x	x		x	-	x		2	B	II
H33	x	x		-	-		x	-	-		3	C	III
H33S	x	x		-	-		x	-	-		3	C	III
NCIMB 1942	-	-		-	-		x	-	-		3	D	III
NCIMB 2035	-	-		-	-		x	-	-		3	D	III

* Please refer to the second figure in the manuscript.

### 2.2. Clustering of *P. luteoviolacea* Strains in Genotypes

**Figure 1:**
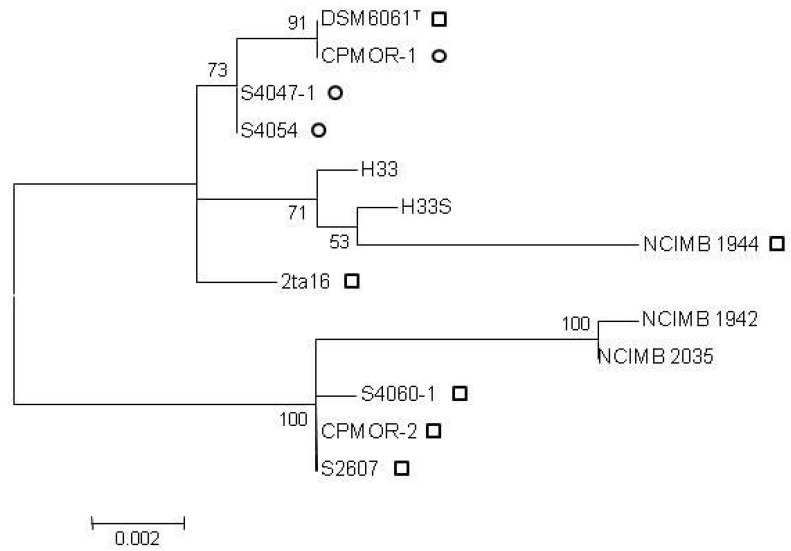
Clustering of 13 *P. luteoviolacea* strains by 16S rRNA gene sequence analysis in MEGA5. Bootstrap values are based on 1000 resamplings. Square: pentabromopseudilin producer, circle: indolmycin producer. All strains produced violacein. Scale bar: substitutions per site.

Phylogenetic trees were created based on 16S rRNA gene sequences alone or concatenated 16S rRNA, *gyrB*, and *recA* gene sequences. The 16S rRNA sequences were identical at a level of >99% and the phylogenetic reconstruction showed no correlation to production of antibiotics within clusters (Figure 1). The resolving power of the 16S rRNA gene is limited when performing phylogenetic analyses below the species level [36] whereas studies have shown the usefulness of housekeeping genes such as *recA* and *gyrB* [37,38,39] in resolving sub-species phylogenetic relations. Seperate analyses of *recA* and *gyrB* genes were carried out to verify that each gene contributed to phylogenetic delineation (data not shown).

**Figure 2:**
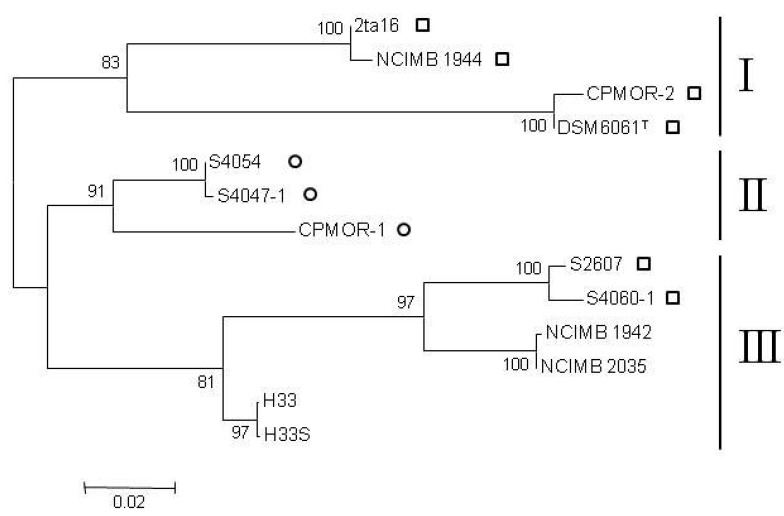
Clustering of concatenated 16S rRNA, *gyrB* and *recA* nucleotide sequences of 13 *P. luteoviolacea* strains analyzed in MEGA5. Bootstrap values are based on 1000 resamplings. The topology was supported by the maximum parsimony method (data not shown). Square: pentabromopseudilin producer, circle: indolmycin producer. All strains produced violacein. Scale bar: substitutions per site.

A phylogenetic analysis of concatenated 16S rRNA, *gyrB* and *recA* genes led to identification of three clusters (Figure 2) as indicated in Table 1 under genotypes. Genotype I represents 4 of the 6 pentabromopseudilin producing strains of chemotype 1 and inhibition profile A. Genotype II is identical to chemotype 2 and inhibition profile B. Genotype III is heterogeneous, covering two strains of chemotype 1 with inhibition profile A, two strains of chemotype 3 with inhibition profile C and two strains of chemotype 3 with inhibition profile D. Within genotype III, sub-clusters tightly reflected associations to chemotype and inhibition profile. 

A correlation among taxonomic units and secondary metabolite synthesis is well established in the world of fungal natural products [40], and was also seen among marine actinomycete *Salinispora* spp. Indeed, within actinobacteria bacterial systematics are emerging as a tool to aid in biodiscovery by focusing on the use of bacterial systematics to dereplicate actinobacterial species in order to focus on novel diversity and its potential for novel chemistry. To our knowledge, this approach has not been tested within the γ-Proteobacteria, or at the infraspecies level within a group of closely related strains as presented here for *P. luteoviolacea*.

### 2.3. MIC of Known Antagonistic Compounds

To investigate the potential role of indolmycin and violacein as antibacterial compounds, the MICs against *V. anguillarum*, *S. aureus,* and the 13 *P. luteovioloacea* strains were determined. Pentabromopseudilin was not included as the compound was unavailable as commercial reference standard. Under the experimental conditions in this study violacein did not inhibit any of the included strains at tested concentrations; hence the MIC of violacein for all tested strains was >128 μg/mL. This is in agreement with the lack of inhibition observed in some violacein producing strains (Table 1). The MIC of indolmycin to *V. anguillarum* was 2 μg/mL whereas *S. aureus* was inhibited by indolmycin at all tested concentrations, resulting in a MIC of ≤0.5 μg/mL. None of the *P. luteoviolaceae*, irrespective of antibiotic profile, were inhibited by indolmycin leading to a MIC of >128 µg/mL. 

Biosynthetic pathways are subjects of horizontal gene transfer events and their organization in genetic clusters facilitates the exchange of entire biological pathways among bacterial strains [41,42,43]. This intuitively supports the common expectation that secondary metabolite production is strain specific [44] and hence bacterial systematics would not be expected to be related to the secondary metabolome. In contrast, distinct species-specific secondary metabolite profiles were observed within the genus *Salinispora* and it was suggested that biosynthetic gene clusters are stably maintained in these species due to the distinct competitive advantage obtained through antibiosis and may in fact represent niche-specific adaptations [45]. Further studies of the biosynthetic pathways behind the antibiotic production of *P. luteoviolacea* and their genomic context are likely to add to our understanding of these concepts. 

## 3. Experimental Section

### 3.1. Bacterial Strains and Culture Conditions

Thirteen *P. luteoviolacea* strains were used in this study (Table 2). They originated from distinct geographical areas and were primarily isolated from surface water or algae. The strains were cultured in a marine minimal medium (MMM) [46] with 4 g/L mannose and 3 g/L casamino acids. All strains were incubated at 25 °C and 200 rpm agitation.

**Table 2:** *P. luteoviolacea* strains used in this study. The phylogenetic position of all strains within the *Pseudoalteromonas luteoviolacea* clade was verified by analysis of partial 16S rRNA gene sequences (>1200 bp).

Strain name	Origin	Source
DSM 6061 ^T^	Mediterranean, Nice	Surface water
S2607	Pacific, Eastern Australia	Rock surface
S4060-1	Pacific, Costa Rica	Seaweed
2ta16	Florida Keys, USA	*M. annularis* coral
CPMOR-2	Mediterranian, Murcia	Surface water
NCIMB1944	Mediterranean, Nice	Surface water
S4047-1	Pacific, Costa Rica	Seaweed
S4054	Pacific, Costa Rica	Seaweed
CPMOR-1	Mediterranean, Murcia	Macroalgae
H33	Sydney, Australia	Unknown
H33S	Sydney, Australia	Unknown
NCIMB1942	Mediterranean, Nice	Surface water
NCIMB2035	Mediterranean, Nice	Surface water

### 3.2. Assays for Inhibition of Bacterial Growth

*Vibrio anguillarum* 90-11-287 [47] and *Staphyloccoccus aureus* 8325 were cultured in tryptic soy broth (Difco, USA). Well diffusion agar assays were carried out as previously described. The assay substrate contained 30 g/L Sea Salts (Sigma, USA) and 10 g/L agar. To support growth of *V. anguillarum* 4 g/L glucose and 3 g/L casamino acids was added. An additional 5 g/L peptone was added to *S. aureus* agar. Antibiosis by live cells was tested by spotting colony mass directly onto the assay plates and observing if clearing zones had formed following incubation for 24 h. 

### 3.3. Analytical Detection of Antibacterial Compounds

Samples for LC-MS analyses were prepared from cultures in MMM extracted with ethyl acetate (EtOAc). Extracts were dried under nitrogen and redissolved in methanol (MeOH). LC-MS samples were analysed using an Agilent 1100 HPLC system with a diode array detector (Waldbronn, Germany) coupled to an LCT TOF mass spectrometer (Micromass, Manchester, UK) using a Z-spray electrospray (ESI) source. A Phenomenex Luna II C_18_ column (50 mm × 2 mm, 3 μm) was used for separation, applying a linear acetonitrile (MeCN)-water (20 mM formic acid) 0.3 mL∙min^−1^ gradient (15%–100%) over 20 min at 40 °C. For all LC-MS analyses, violacein and indolmycin were detected in positive ionisation mode (ESI^+^), while pentabromopseudilin was detected in negative mode (ESI^−^).

### 3.4. PCR Amplification and Sequencing

DNA was purified from overnight *P. luteoviolacea* cultures using the NucleoSpin Tissue kit (Machery-Nagel, Germany) or QIAGEN Genomic-Tip G/100 (QIAGEN, USA) following the manufacturer’s protocol. 16S rRNA genes from strains H33, H33S, NCIMB 1942, NCIMB 1944 and NCIMB 2035 and *gyrB* and *recA* genes from all strains were amplified by PCR. One reaction consisted of 2.5 µL 10× Hot Start PCR buffer (Fermentas, Canada), 2.5 µL 2 mM dNTP mix, 4 µL 25 mM MgCl_2_, 0.8 µL 12.5 µM forward primer, 0.8 µL 12.5 µM reverse primer, 12.28 µL MilliQ H_2_O, 0.2 µL Maxima Hot Start *Taq* DNA polymerase (Fermentas, Canada) and 1 µL DNA template at 50 ng/µL for a total volume of 25 µL. The reactions were performed on an Applied Biosystems Veriti 96 well cycler. 16S rRNA genes were amplified according to. The primers and reaction conditions used for *gyrB* amplification were as described in [48]. Primers and conditions for amplification of *recA* fragments were according to [49]. Sequencing was done by Eurofins MWG Operon, Germany. Nucleotide sequences generated in this study were deposited in GenBank under accession numbers: 16S rRNA JQ250820-JQ250824, *gyrB* JQ280430-JQ280442 and *recA* JQ280417-JQ280429. Accession numbers for 16S rRNA nucleotide sequences previously available from GenBank: NR_026221, FJ457234, FJ457187, FJ457230, FJ457238, EU158365, EU158366 and FJ952782.

### 3.5. Phylogenetic Analysis

Analysis of concatenated gene sequences, termed multi locus sequence analysis, has succesfully been used to infer robust phylogenies within, e.g., the *Vibrionaceae* [49], and a similar approach was used in this study. The 16S rRNA gene sequences were obtained from GenBank or by sequencing and aligned in MEGA5 using MUSCLE [50]. The evolutionary history was inferred using the maximum likelihood method based on the Kimura 2-parameter model [51]. The phylogenetic tree was constructed using MEGA5 default settings. For analysis of *recA* and *gyrB* sequences, an alignment was created for each gene in MEGA5 using MUSCLE. The alignments were curated manually and trimmed to be of equal length and in-frame. 16S rRNA, *gyrB* and *recA* alignments were concatenated and phylogenetic analyses were performed in MEGA5. Evolutionary relationships based on the nucleotide sequences were inferred using the maximum likelihood method with the general time reversible model [52], assuming a gamma distributed substitution rate with five discrete categories. Neighbor-joining phylogenetic trees were generated and tested with 1000 bootstrap replications. 

### 3.6. Minimum Inhibitory Concentrations (MIC) of Violacein and Indolmycin

MICs of commercially available violacein (Sigma, USA) and indolmycin (Bioaustralis, Australia) standards to *P. luteoviolacea* strains, *V. anguillarum* 90-11-287, and *S. aureus* 8325 were tested. MIC assays were carried out in 96-well microtiter plates according to the guidelines by the clinical and laboratory standards institute [53], with minor modifications. *P. luteoviolacea* was cultured in MMM with 4 g/L mannose and 3 g/L casamino acids, *V. anguillarum* in MMM with 4 g/L glucose and 3 g/L casamino acids, and *S. aureus* in MHB. Overnight bacterial cultures were diluted to 10^5^ CFU/mL and 90 µL added per well. 10 µL of antibiotic solution was added to each well. The antibiotics were tested in serial two-fold dilutions at final concentrations of 0.5 µg/mL to 128 µg/mL. Controls were included for no antibiotic and ethanol solvent. The well containing the lowest concentration of antibiotic that had no visual bacterial growth after 48 h corresponded to the MIC.

## 4. Conclusions

In summary, combining simple inhibitory screening and information on gene based sub-types allows a targeted biological dereplication of *P. luteoviolacea* strains before chemical analysis of secondary metabolite production. This approach potentially enables selection of key strains and a reduction of the bottle neck and expenses associated with screening of large collections of non-dereplicated bacterial strains. 
